# Effects of Brain Breaks Video Intervention of Decisional Balance among Malaysians with Type 2 Diabetes Mellitus: A Randomised Controlled Trial

**DOI:** 10.3390/ijerph18178972

**Published:** 2021-08-26

**Authors:** Aizuddin Hidrus, Yee Cheng Kueh, Bachok Norsa’adah, Yu-Kai Chang, Garry Kuan

**Affiliations:** 1 Biostatistics and Research Methodology Unit, School of Medical Sciences, Universiti Sains Malaysia, Kubang Kerian 16150, Kelantan, Malaysia; aizuddinh88@gmail.com; 2 Community and Family Medicine Department, Faculty of Medicine and Health Science, Universiti Malaysia Sabah, Kota Kinabalu 88400, Sabah, Malaysia; 3 Department of Physical Education and Sport Sciences, National Taiwan Normal University, Taipei 106, Taiwan; yukaichangnew@gmail.com; 4 Institute of Research Excellence in Learning Science, National Taiwan Normal University, Taipei 106, Taiwan; 5 Exercise and Sports Science, School of Health Sciences, Universiti Sains Malaysia, Kubang Kerian 16150, Kelantan, Malaysia; 6 Department of Life Sciences, Brunel University London, Uxbridge UB8 3PH, UK

**Keywords:** Brain Breaks^®^, video exercise, decisional balance, diabetes mellitus, physical activity, repeated measures

## Abstract

Brain Breaks^®^ are structured physical activity (PA) web-based videos designed to promote an interest in learning and health promotion. The objective of this study was to examine its effects on decision balance (DB) which consists of the perceived benefits (Pros) and perceived barriers (Cons) of exercise in people with type 2 diabetes mellitus (T2DM). A randomised controlled trial was conducted among people with T2DM at Hospital Universiti Sains Malaysia. The intervention group received Brain Breaks videos for a period of four months. The intervention and control groups completed the validated Malay version of DB questionnaire for five times, at pre-intervention, the first month, the second month, the third month, and post-intervention. Multivariate Repeated Measures Analysis of Variance was performed for data analysis. A total of 70 participants were included (male = 39; female = 31) with a mean age of 57.6 years (SD = 8.5). The intervention group showed a significant change in the Pros and Cons factors of DB scores over time. The intervention group showed significantly higher scores for the Pros (*p*-value < 0.001) and lower scores for the Cons (*p*-value = 0.008) factors than the control group. In conclusion, the Brain Breaks video is an effective intervention to improve decisional balance in patients with T2DM to help them in deciding on behaviour change to be more physically active.

## 1. Introduction

The Centers for Disease Control and Prevention (CDC) [1] defined diabetes as “a chronic (long-lasting) health condition that affects how your body turns food into energy”. From a different perspective, diabetes is defined by the National Institute of Diabetes and Digestive and Kidney Diseases (NIDDK) [2] as “a disease that occurs when your blood glucose, also called blood sugar, is too high”. According to a recent International Diabetes Federation (IDF) [3] report, global diabetes prevalence was 463 million in 2019 and is expected to rise to 578 million in 2030 and 700 million in 2045. From the total of 463 million, 163 million are from the Western Pacific region, 88 million are from the South-East Asia region, 59 million are from Europe, 55 million are from the Middle East and North Africa, 48 million are from North America and the Caribbean, 32 million are from South and Central America, and 19 million are from the Africa region. The IDF also reported that diabetes affects people of all ages, typically showing higher prevalence with increasing age, up to 60–69 years.

Type 2 diabetes mellitus (T2DM) and its complications have made an enormous contribution worldwide to the burden of death and incapacity. More than 90% of diabetes mellitus cases are T2DM [4]. The early stage of T2DM pathogenesis are insulin resistance and dysfunction of β-cells that leads to insulin release reduction [5]. T2DM comprises a range of hyperglycaemic dysfunctions that are a result of combining insulin resistance, insufficient insulin secretion, and excessive and inadequate glucagon secretion [6]. Khardori [6] added, that worsened T2DM is associated with an array of neuropathic, macrovascular, and microvascular complications.

In the Malaysian population, more than a decade ago, Mustaffa [7] statistically showed the development of diabetes until it became an epidemic in Malaysia. Diabetes prevalence increased from 0.65% in 1960 to 2–4% in the early 1980s [7]. In the same research, it was also stated that in the mid-1990s, the prevalence rose up to 8–12% and the percentage as predicted increased in 1998 with a reported 14% of the prevalence appearing. As we all know, diabetes potentially causes complications for patients if it is not well controlled. According to Mustaffa [7], diabetic patients have been complicated by reported retinopathy (53%), neuropathy (58%), and microalbuminuria (52%). He then added that Malaysian diabetics were at the high potential of suffering from ischaemic heart disease and stroke as complications of macrovascular. The macrovascular complications were due to late diagnosis and poor glycaemic control (mean HbA1c > 9%), and also due to the close relation to obesity (43–52% are either overweight or obese, more in female Malays and Indians), hypertension (10–37%) and hyperlipidaemia (63–76%).

For many years, physical activity (PA) and exercise have been empirically accepted by clinicians and researchers which can improve the health status of patients with any kind of disease. For example, a study conducted by Taylor et al. [8], found that coronary heart disease patients who were given exercise training (intervention group) showed a decrease in the percentage of total and cardiac mortality rates, 20% and 26%, respectively, compared to the regular medical care control group. Other than treating existing diseases, PA could be adopted as a prevention method. Lynch, Neilson and Friedenreich [9] conducted a review of 73 epidemiological studies of PA and breast cancer risk and concluded that the most physically active female group has a 25% lower risk of breast cancer than the least active female group.

Changing lifestyle, including weight loss, increased PA and healthy diet, continues to be one of the top-of-the-line T2DM management strategies [5]. An randomised controlled trial (RCT) was done in the USA on the health benefits of aerobic and resistance training in individual with diabetes, researchers concluded that in the group undertaking combined aerobic and resistance training, after the nine months of training, HbA1c levels were reduced [10]. It indicates that combining both aerobic and strength exercises is more advantageous than performing just one type of exercise if time is limited [11]. In addition, an extra-virgin olive oil enriched Mediterranean diet might prevent diabetic retinopathy, however not diabetic nephropathy [12].

The decisional balance is a Transtheoretical Model psychological construct that comprises benefits (Pros) and drawbacks (Cons) of maintaining current behaviour or beginning a new behaviour [13]. It can also be defined as a multidimensional collection of ideals viewed as advantages and disadvantages correlated with behavioural change [14]. After considering the benefits (Pros) and disadvantages (Cons) of practice, people prefer to change their own practice. According to people who effectively alter new behaviour, their advantages will change rather than drawbacks and their advantages should look more than disadvantages [15]. Benefits for health such as stress relief, better sleep patterns, and more energy and stamina are examples of Pros for exercise, while examples of Cons are injuries, time constraints, and bad weather.

Living in a technologically empowered world, we cannot neglect the use of media influence on education and human development. This effect may be positive as well as negative. As previously stated, the Brain Breaks video is a web-based structured PA break that promotes an individual’s health and learning, and it has recently emerged as a new promising intervention introduced by HopSports [16]. It has been commonly applied among school students as part of their physical education [17,18,19,20,21,22]. Brain-breaks have always been adopted in research with PA intervention settings where participants are divided into at least two groups.

Although the Brain Breaks intervention has been widely adopted, most of the studies only focus on school students. As the Brain Breaks intervention is categorised as a PA intervention, its use should be applied more among other populations such as university students, healthy adults or patients who are suffering from any kind of disease. For the time being, the researchers only discovered one brain breaks intervention study conducted in people with T2DM, focusing on motives to PA [23]. As one of the PA interventions, the researchers thought it was still relatively new to the Malaysian population. As a result, the researchers decided to use it as an intervention material in the current study at early exposure, and it could benefit the Malaysian population, particularly those suffering from diseases that require PA as part of their self-management.

## 2. Materials and Methods

### 2.1. Study Design, Recruitment, and Sampling

A RCT was conducted in the hospital of Universiti Sains Malaysia (HUSM), Kelantan, Malaysia. The present study focuses on people with T2DM, and we employed purposive sampling to recruit participants. The people with T2DM were briefed about the study by the researchers. Participants who agreed to volunteer for the study were randomly allocated to the intervention and control groups using block randomisation [24]. Through block randomisation, the researcher developed a block to similarly assign sample numbers and each group were assigned with block numbers [25]. As the present study have only two groups, intervention and control, hence two blocks (AB and BA) randomisation were applied. Hence, from the 100 participants, both groups were assigned equal numbers of participants, *n* = 50. The inclusion criteria include 18 years and above Malaysians who were clinically diagnosed with T2DM, can read and understand the Malay language, also, understand and agree the explained information to participants by the researchers. While for exclusion criteria, those who have disabilities that prevent them from being physically active.

### 2.2. Instruments

There are two sections of the self-administered questionnaire, (1) the demographic details, and (2) the Malay version of Decisional Balance (DB-M) scale. For the demographic details, information such as age (years), gender, and ethnicity were collected from this section.

The Decisional Balance (DB) scale is a questionnaire that consists of 10 items which were initially developed by Plotnikoff et al., (2001) [13]. Each item was measured by using a 5-point Likert scale, from 1 (not at all confident) to 5 (extremely confident). There are two factors in DB, Pros that represent the positive aspect of an individual’s behavioural changes, and Cons represent the negative aspect. When it comes to exercise, the decisional balance represents perceived benefits (Pros) and perceived barriers (Cons) [26]. Self-confidence, physical strength, and aerobic ability and cited as perceived benefits of exercise; whereby, physical discomfort and financial concerns are cited as perceived barriers to exercise [26]. The Pros factor consists of five items and there are: physical activity would help me reduce tension or manage stress, I would feel more confident about my health by getting physical activity, I would sleep better, physical activity would help me have a more positive outlook, physical activity would help me control my weight. The Cons factor consists of five items and there are: I am too tired to get physical activity because of my other daily responsibilities, physical activity would take too much of my time, I would have less time for my family and friends if I participated in physical activity, I’d worry about looking awkward if others saw me being physically active, getting physical activity would cost too much money. The researchers adopted the DB-M scale that has been validated by Kuan, Sabo, Sawang and Kueh [26]. The DB-M scale has good validity and reliability. Based on the confirmatory factor analysis results, the DB-M model fit the data well with acceptable fit indices (CFI = 0.979, TLI = 0.969, RMSEA = 0.047, and SRMR = 0.037). The internal consistency reliability was excellent with a Cronbach’s alpha value of 0.86 for both the Pros and Cons subscales. It also showed good test-retest reliability results with an intraclass correlation value of 0.98.

### 2.3. Sample Size Determination

The sample size was estimated for time (within factor), group (between factor) and interaction (within-between) effects. Using the GPower 3.1, sample size calculation software, with effect size = 0.25 (medium effect) [27], type I error = 0.05, power = 0.8, number of groups = 2, number of measurements = 5, and expected correlation among repeated measure = 0.5, the total sample size calculated was 78 for between factor, 22 for within factor, and 22 within-between interaction. Thus, the largest sample size was 78 for this study with 36 participants per group. However, after a thorough discussion, the researcher decided to recruit 100 participants (50 per group) for this study as a precaution against the high withdrawal rate from the participants.

### 2.4. Procedure

At baseline or before the intervention started, both groups (intervention and control) were required to answer on the DB-M scale. Then, participants in the intervention group were invited to a WhatsApp group where the Brain Breaks videos were given during the period of the intervention phase. In this period (four months), exercise videos with ten minutes duration specifically designed for diabetes patients were uploaded into the WhatsApp group. All the participants in the intervention group were required to perform the exercise either outdoors or indoors. The videos were uploaded to the WhatsApp group weekly as a regular reminder for the participants. On the first day of each week, different exercise videos were given to avoid participants from getting bored with the same exercise. As part of that, every participant in the intervention group was given an adherence logbook for the purpose of progress monitoring. Participants were required to report to the researcher about their progress of exercise based on the logbook they have noted down. At the end of the intervention, the participants were required to return their adherence logbook to the researcher for assessment. As for the control group, a brochure with the benefits of PA for their health was given to the participants. They did not receive Brain Breaks videos and were not required to perform the exercise. However, they would receive the same intervention videos that were given to the intervention group at the end of the study. Thus, they would get the same benefits similar to the intervention group in the future. The duration of the intervention was four months. At the end of each month, participants in both the intervention and control groups were required to answer the DB-M. The outcome of the study was based on the score of the DB-M.

This study obtained approval from the USM Human Research Ethics Committee (USM/JEPeM/18040201) and was conducted in accordance with the guidelines of the International Declaration of Helsinki. Before the study began, participants were informed that their participation was voluntary and that they could withdraw at any time without incurring any loss or penalty. Written informed consent was obtained from each participant before they participated in the study. This study was registered under the clinical trial of the ISRCTN registry, which is recognized by the World Health Organization (registry number: ISRCTN14952589).

### 2.5. Data Analysis

The Statistical Package for the Social Sciences (SPSS) version 26.0 was used in conducting the data analysis. The data consisted of two groups (i.e., intervention and control) with a five-time measurement of the outcome variables (score of the DB-M). The effect of the Brain Breaks video exercises on decisional balance, which consists of Pros and Cons, were investigated using repeated measures multivariate analysis of variance (RM MANOVA). The effects examined consisted of time, group, and interaction (time ∗ group) effects. Mauchly’s test of sphericity was used to test whether the sphericity assumption was met. If the assumption is violated, the F-statistic based on Greenhouse-Geisser is reported. A *p*-value of <0.05 was taken as a significant result.

## 3. Results

### 3.1. Participants

**Figure 1:**
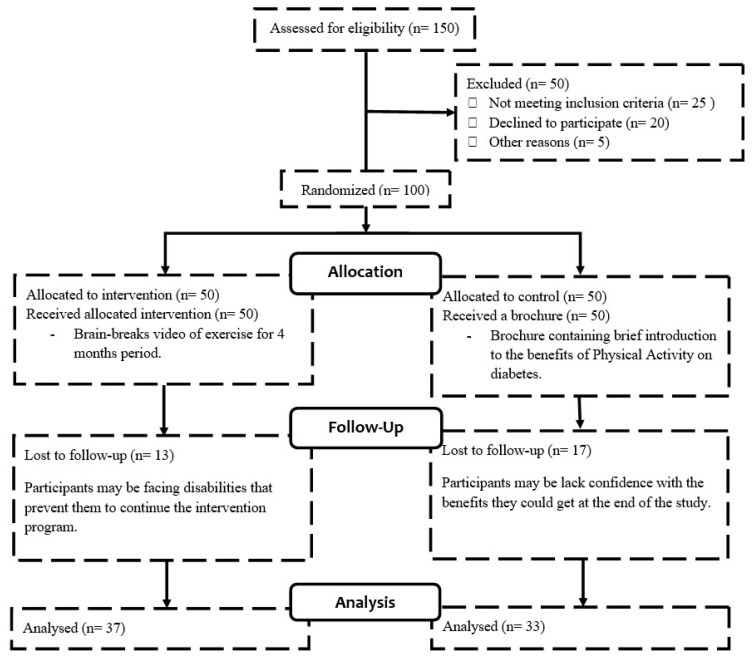
Flow chart of participant’s group allocation/randomisation.

The randomisation included 100 T2DM patients. This produced 50 members for each group (intervention and control). In the middle of the intervention period, however, 13 intervention members and 17 control participants withdrew for personal reasons. Therefore, 37 participants in the intervention group and 33 participants in the control group with complete data were obtained at the end of the study. Figure 1 shows the participants’ group allocation in this study. Table 1 presents the demographic information for T2DM patients.

### 3.2. Results of RM MANOVA

The researcher started the analysis by checking the assumption of the compound symmetry of the data. Mauchly’s test was used to check the compound symmetry assumption. From the result, the *p*-value produced for both factors were significant (*p*-value < 0.05) indicated that the assumption of compound symmetry was not met. Hence, multivariate test statistics result was referred for time effect. The researcher decided to proceed with multivariate test statistics as the assumption has been violated with the Pillai’s Trace [F-stat(df) = 7.730 (8, 552), *p*-value < 0.001] and Wilk’s Lambda [F-stat(df) = 8.125 (8, 550), *p*-value < 0.001] both produced significant *p*-value.

Then, pairwise comparison with confidence interval adjustment was carried out to determine the differences within the group. Table 2 below show the score comparison within both groups (intervention and control) based on time (Time effect).

The intervention group produced a significant mean score difference in the DB-M scale’s Pros and Cons factors, as well as time comparisons. The Intervention group also shows an increasing trend in Pros but a decreasing trend in Cons over time. In the control group, there were a few time comparisons that resulted in a non-significant mean score difference, such as the 3rd month-Post in the Pros factor and the 1st month-2nd month in the Cons factor. There was also a decreasing trend in scores over time in the control group for the Pros and an increasing trend for the Cons.

The overall mean score of both groups (group effect) was compared and the results are in Table 3. Based on the results, the overall mean score of the DB-M scale between both groups was significantly different. Furthermore, the intervention group had a higher mean score for the Pros factor than the control group, and vice versa for the Cons factor.

**Figure 2:**
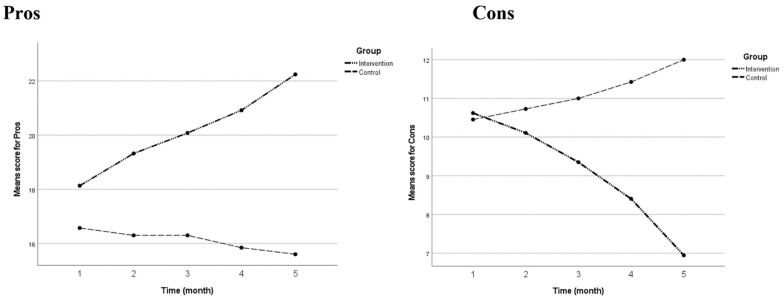
Mean score changes for Pros and Cons of DB-M from pre-intervention to post-intervention. Note the plot shows the mean score of Pros and Cons of DB-M scores (y-axis) for pre-intervention (time 1), 1st month (time 2), 2nd month (time 3), 3rd month (time 4) and post-intervention (time 5; x-axis) for both groups (intervention and control).

Based on multivariate test statistics, there was a significant interaction effect between time and group (Time ∗ Group effect), with Pillai’s Trace [F-stat(df) = 35.920 (8, 544), *p*-value < 0.001] and Wilk’s Lambda [F-stat(df) = 52.750 (8, 542), *p*-value < 0.001] both produced significant *p*-value. The comparison of the mean score for Pros and Cons between the two groups based on within-between groups (Time ∗ Group effect) for each time point are shown in Table 4. For factor Pros, the intervention and control groups showed non-significant differences in the mean score at pre-intervention. However, a significant mean difference between both groups was observed starting from 1st month until post-intervention. For factor Cons, the intervention and control groups showed a non-significant difference in the mean score at pre-intervention and 1st month. However, a significant mean difference between both groups was observed starting from the 2nd month until post-intervention. The changes from pre- to post-intervention among the two groups can be observed in Figure 2.

## 4. Discussion

The main objective of the present study was to determine the effect of Brain Break exercise videos on the changes in the DB of T2DM patients in terms of perceived Pros and Cons. The effect of Brain Break exercise videos on DB was measured based on three main components, which were time, group, and interaction (time ∗ group). For the time effect, all the measurement time comparisons were significant for both DB components of Pros and Cons in the intervention group. While for the control group, there were no significant differences in the measurement time for both DB components Pros (e.g., 2nd month–3rd month) and Cons (e.g., 1st month–2nd month). For the group effect, results showed a significant overall mean score difference between the two groups on both DB components of Pros and Cons. A significant interaction effect was observed for both DB components of Pros and Cons which indicated that the magnitude and direction of changes from pre- to post-intervention were different between the intervention and the control groups.

Results of the present study displayed similarity with a study conducted by Moeini et al. [28]. Moeini et al. [28] performed a quasi-experimental intervention study among employees of the defensive industry. The objective of the study was to increase PA among the employees as one of the most effective ways to reduce the risk of non-communicable diseases. A total of 60 employees with the age range from 20 to 57 years old were recruited using simple random sampling. They were divided into intervention and control groups that required them to complete the questionnaires before and three months after the intervention. Only the intervention group received the educational programs during the intervention period. The physical capacity score measured by the Ergo-meter bicycle showed significant improvement in the intervention group (*p*-value = 0.016) at the end of the study. Total DB scores were also significantly higher in the experimental group (*p*-value < 0.001) compared to the control group. A conclusion of TTM based educational programs/interventions were beneficial for physical capacity and PA enhancement was made [28].

In addition, PA was also found to be related to DB in a study done by Shtaynberger and Krebs [29], who performed a study of the adult cancer survivorship population in New York. Participants were a total of 86 completed primary treatment of breast/prostate cancer patients. One of the study objectives was to assess the relationship between DB and PA. The findings presented a significant relationship between the Total Metabolic Equivalent of Task units (METs) with both Pros (*p*-value = 0.012) and Cons (*p*-value = 0.003).

While the present study shows a positive result, it is necessary to highlight limits and weaknesses. Due to the time constraints facing by the researchers, participants were recruited from only one hospital in Malaysia. The participation of people with T2DM from more hospitals in Malaysia could have contributed to better results in this study. In the future, a multicentre community trial should therefore be used to obtain more responses from different hospitals. Furthermore, the researchers were unable to closely follow the commitment of the participants in the intervention other than with a logbook. Participants may have dishonest or misrepresented their participation in the intervention. This was unavoidable as most patients with T2DM were difficult to assemble in a location where the intervention could be observed every day as a result of logistical and transport constraints. However, we regularly send reminders to the WhatsApp group at daily intervals to ensure the participants adhere to the intervention.

Another limitation is the quantitative methods that need to be used to measures the variables among participants. Respondent bias, acquiescence bias, demand characteristics, extreme responding, and social desirability bias could happen as the participants could answer the questionnaire dishonestly and insincerely. These biases may give a negative impact on the reliability of the completed questionnaire from the participants. However, the researcher had regularly encouraged the participants to answer the questionnaire sincerely and only according to what they were thinking and their current condition.

Another limitation is that only those T2DM patients with internet services on mobile phones were able to participate in the study. This was necessary for the participants to install the WhatsApp application for sharing Brain Breaks videos. We were unable to recruit more participants due to this issue and time constraints. But the researchers believe that this was the best way to get participants involved after taking the logistics and transport restrictions into account. Finally, this study only focuses on a single non-communicable disease (T2DM). Therefore, we urge more kinds of non-communicable diseases to be included in future studies to present broader results.

We believe, however, that the effective design of the study was a community test with block randomisation, which strengthened this study sufficiently to produce good results. Randomisation blocks have been used to reduce selection distortions so that the current study is more confidential for future references. Further, during the trial period (for monitoring purposes), the researchers conducted three repeated measurements and regularly followed up with the participants through WhatsApp to make sure that they adhered to the intervention. The other two repeated measurements (pre- and post-intervention) were conducted through face-to-face meeting with the participants. Finally, RM MANOVA was carried out for this study rather than multiple repeated measures analysis of variance (RM ANOVA). It was due to MANOVA has greater power and improves the chances of detecting differences between groups than ANOVA [30]. Furthermore, the sphericity assumption in RM MANOVA allows you to control the type of error [31], making them superior to the Paired *t*-test. With proper choice of statistical analysis, the results yielded should be more accurate and more reliable.

## 5. Conclusions

From the results, the researchers conclude that the brain-breaks intervention is an effective intervention to improve participants’ decision-making skills. Participants who were in the intervention group showed significant improvement in the DB mean score compared to participants in the control group. At the end of the study, the intervention group demonstrated superior decision-making abilities, with a higher mean score for both the Pros and Cons of DB than the control group. The intervention with the Brain Breaks videos also helped to demonstrate differences in the trends in the mean scores between the two groups. The intervention group had an uptrend in the Pros factor and a downtrend in the Cons factor. The opposite trends between the two factors indicate that, at the end of the study, the intervention group was more focused on the positive impact and benefits of PA rather than the negative impacts and obstacles of performing PA. In the control group, however, the opposite occurred.

## Figures and Tables

**Table 1:** Participants demographic characteristics.

Characteristics	Intervention				Control				*p*-Value
	Frequencies	Percentage	Median (IQR)	Mean (SD)	Frequencies	Percentage	Median (IQR)	Mean (SD)	
**Gender**									0.208 ^a^
Male	18	48.6%			21	63.6%			
Female	19	51.4%			12	36.4%			
**Age**			56.00 (10.00)				63.00 (8.00)		<0.001 ^c^
**Ethnicity**									0.242 ^b^
Malay	34	91.9%			29	87.9%			
Chinese	3	8.1%			1	3.0%			
Indian	0	0%			1	3.0%			
Others	0	0%			2	6.1%			
**HbA1c (*n* = 65)**				80.11 (3.74)				76.23 (4.26)	0.529 ^d^
**BMI**			28.06 (5.90)				26.59 (6.19)		0.902 ^c^

Note: ^a^ Pearson Chi-square test, ^b^ Fisher exact test, ^c^ Mann-Whitney test, ^d^ Independent *t*-test.

**Table 2:** Comparison of DB-M score within group based on time (Time effect).

Comparison	Intervention Group				Control Group			
	Pros		Cons		Pros		Cons	
	MD (95% CI)	*p*-Value	MD (95% CI)	*p*-Value	MD (95% CI)	*p*-Value	MD (95% CI)	*p*-Value
Pre-1st month	−1.19 (−1.68, −0.70)	<0.001	0.51 (0.14, 0.89)	0.002	0.27 (0.002, 0.54)	0.048	−0.27 (−0.60, 0.06)	0.176
Pre-2nd month	−1.95 (−2.60, −1.29)	<0.001	1.27 (0.65, 1.89)	<0.001	0.27 (0.002, 0.54)	0.048	−0.55 (−1.00, −0.09)	0.010
Pre-3rd month	−2.78 (−3.80, −1.77)	<0.001	2.22 (1.21, 3.23)	<0.001	0.73 (0.11, 1.35)	0.012	−0.97 (−1.52, −0.42)	<0.001
Pre vs. Post intervention	−4.10 (−5.40, −2.81)	<0.001	3.68 (2.10, 5.25)	<0.001	0.97 (0.10, 1.84)	0.019	−1.55 (−2.27, −0.83)	<0.001
1st month–2nd month	−0.76 (−1.05, −0.46)	<0.001	0.76 (0.33, 1.18)	<0.001	-	-	−0.27 (−0.57, 0.03)	0.102
1st month–3rd month	−1.60 (−2.28, −0.91)	<0.001	1.70 (0.90, 2.50)	<0.001	0.46 (−0.06, 0.96)	0.113	−0.70 (−1.14, −0.25)	<0.001
1st month–Post	−2.92 (−3.88, −1.96)	<0.001	3.16 (1.81, 4.51)	<0.001	0.70 (−0.003, 1.40)	0.052	−1.27 (−1.85, −0.70)	<0.001
2nd month–3rd month	−0.84 (−1.36, −0.31)	<0.001	0.95 (0.42, 1.48)	<0.001	0.46 (−0.06, 0.96)	0.113	−0.42 (−0.69, −0.16)	<0.001
2nd month-Post	−2.16 (−2.94, −1.38)	<0.001	2.41 (1.35, 3.46)	<0.001	0.70 (−0.003, 1.40)	0.052	−1.00 (−1.46, −0.55)	<0.001
3rd month-Post	−1.32 (−1.76, −0.89)	<0.001	1.46 (0.75, 2.17)	<0.001	0.24 (−0.17, 0.66)	0.882	−0.58 (−1.03, −0.12)	0.006

Notes. Repeated measure MANOVA between-group analysis with regard to time was applied, 95% CI = 95% confidence interval.

**Table 3:** Overall mean differences of DB-M score among two groups.

Comparison (Intervention and Control Groups)	Mean Difference (95% CI)	F (df)	*p*-Value
Pros	4.01 (2.63, 5.40)	33.447 (1, 68)	<0.001
Cons	−2.04 (−3.52, −0.55,)	7.489 (1, 68)	0.008

Notes. Repeated measures MANOVA between group was applied, 95% CI = 95% confidence interval.

**Table 4:** Comparison of the mean score for Pros and Cons from the DB-M scale among two groups based on time (Time ∗ Group effect).

Factor	Time	Group	Mean (95% CI)	*p*-Value
Pros	Pre-intervention	Intervention	18.14 (16.95, 19.32)	0.075
		Control	16.58 (15.32, 17.83)	
	1st month	Intervention	19.32 (18.27, 20.38)	<0.001
		Control	16.30 (15.19, 17.42)	
	2nd month	Intervention	20.08 (19.06, 21.11)	<0.001
		Control	16.30 (15.22, 17.39)	
	3rd month	Intervention	20.92 (20.06, 21.78)	<0.001
		Control	15.85 (14.94, 16.76)	
	Post-intervention	Intervention	22.24 (21.46, 23.03)	<0.001
		Control	15.61 (14.77, 16.44)	
Cons	Pre-intervention	Intervention	10.62 (9.32, 11.92)	0.861
		Control	10.46 (9.08, 11.83)	
	1st month	Intervention	10.11 (8.92, 11.30)	0.478
		Control	10.73 (9.47, 11.99)	
	2nd month	Intervention	9.35 (8.29, 10.42)	0.038
		Control	11.00 (9.87, 12.13)	
	3rd month	Intervention	8.41 (7.48, 9.33)	<0.001
		Control	11.42 (10.45, 12.40)	
	Post-intervention	Intervention	6.95 (6.18, 7.72)	<0.001
		Control	12.00 (11.19, 12.82)	

Note. Repeated measures MANOVA within the group was applied, MD = mean difference, 95% CI = 95% confidence interval.

## Data Availability

The data is available upon request from the authors.
